# Editorial: Severe fever with thrombocytopenia syndrome

**DOI:** 10.3389/fmicb.2023.1281600

**Published:** 2023-09-05

**Authors:** Li-Fen Hu, Yun-Jia Ning, Jia-Bin Li

**Affiliations:** ^1^Department of Infectious Diseases, The First Affiliated Hospital of Anhui Medical University, Hefei, Anhui, China; ^2^Anhui Province Key Laboratory of Infectious Diseases, Anhui Medical University, Hefei, China; ^3^Institute of Bacterial Resistance, Anhui Medical University, Hefei, China; ^4^Wuhan Institute of Virology and Center for Biosafety Mega-Science, Chinese Academy of Sciences, Wuhan, China; ^5^Hubei Jiangxia Laboratory, Wuhan, China

**Keywords:** severe fever with thrombocytopenia syndrome, antibody, invasive pulmonary aspergillosis, immunity, *Dabie bandavirus*

Severe fever with thrombocytopenia syndrome (SFTS) is an emerging infectious disease caused by a novel bunyavirus, which was first isolated from China in 2010. This was an RNA virus belonging to *Bunyavirales* order, *Pheniviridae* family, *Bandavirus* genus which was renamed as *Dabie bandavirus* (DBV) by the International Committee on Taxonomy of Viruses in 2019. SFTS is increasingly becoming a public health threat due to its high morbidity and mortality. The DBV pathogenesis and severity of the disease are not yet clear. Besides, there are no specific treatment and preventive measures against DBV. More research should be focused on severe fever with thrombocytopenia syndrome.

The objective of this topic was to encourage original research about pathogenesis of multiple organ dysfunction syndromes and immune responses elicited by DBV, and secondary infection contribution to case fatality in SFTS patients such as bacterial and fungal infections, especially invasive pulmonary aspergillosis. After rigorous peer review, a total of four articles have been published in this Research Topic. Jin et al. mentioned that tripartite motif-containing 3 (TRIM3), as a member of the TRIM protein family can inhibit the production of cytokines by regulating the degradation of TLR3 through K48-linked ubiquitination, which can be a therapeutic target for improving the prognosis of SFTS. Dai et al. reported that smoking history, cough, creatinine, admission to ICU, broad-spectrum, and corticosteroid therapies were the independent risk factors for invasive pulmonary aspergillosis (IPA) in SFTS patients. There is a strong dose-dependent association between smoking and IPA development in SFTS patients. They emphasized that prophylactic antifungal therapy should be considered for SFTS patients with these risk factors. Chen et al. mentioned that Ferritin and PCT levels, especially ferritin, could be potential inflammatory biomarkers for predicting the prognosis of patients with SFTS in its early stages. Liang et al. showed a consistent rise in the incidence of SFTS over the past 12 years, accompanied by a relatively high case fatality rate, making it a critical public health issue. All the articles focused on the research about severity of the disease, which may be helpful for disease condition evaluation and treatment of SFTS patients.

## Author contributions

L-FH, Y-JN, and J-BL wrote the original draft and reviewed and edited the Editorial. All authors contributed to the article and approved the submitted version.

